# Transition to turbulence in Taylor-Couette ferrofluidic flow

**DOI:** 10.1038/srep10781

**Published:** 2015-06-12

**Authors:** Sebastian Altmeyer, Younghae Do, Ying-Cheng Lai

**Affiliations:** 1 Institute of Science and Technology Austria (IST Austria), 3400, Klosterneuburg, Austria; 2 Department of Mathematics, KNU-Center for Nonlinear Dynamics, Kyungpook National University, Daegu, 702-701, South Korea; 3 School of Electrical, Computer and Energy Engineering, Arizona State University, Tempe, Arizona, 85287, USA

## Abstract

It is known that in classical fluids turbulence typically occurs at high Reynolds numbers. But can turbulence occur at low Reynolds numbers? Here we investigate the transition to turbulence in the classic Taylor-Couette system in which the rotating fluids are manufactured ferrofluids with magnetized nanoparticles embedded in liquid carriers. We find that, in the presence of a magnetic field transverse to the symmetry axis of the system, turbulence can occur at Reynolds numbers that are at least one order of magnitude smaller than those in conventional fluids. This is established by extensive computational ferrohydrodynamics through a detailed investigation of transitions in the flow structure, and characterization of behaviors of physical quantities such as the energy, the wave number, and the angular momentum through the bifurcations. A finding is that, as the magnetic field is increased, onset of turbulence can be determined accurately and reliably. Our results imply that experimental investigation of turbulence may be feasible by using ferrofluids. Our study of transition to and evolution of turbulence in the Taylor-Couette ferrofluidic flow system provides insights into the challenging problem of turbulence control.

In classical Newtonian fluid, it is known that turbulence typically occurs in the high Reynolds number regime^1^,^2^. Here, the Reynolds number (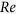) is a dimensionless quantity defined as 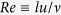, where 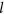 and 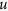 are the characteristic length scale and a typical velocity of the flow in a particular geometry, respectively, and 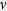 is the kinematic viscosity. The quantities *l* and *u* may differ essentially from the size of the streamline body and the mean velocity around, respectively. As a result, for different flow systems the critical *Re* values of transition to turbulence can be quite different and, in some cases (e.g., pipe flows), may even approach infinity^3^. In the paradigmatic setting of a uniform flow of velocity *u* past a cylinder of diameter *l*, when *Re* is of the order of tens, the flow is regular. For *Re* in the hundreds, von Kármán vortex street forms behind the cylinder^4^, breaking certain symmetries of the system. Fully developed turbulence, in which the broken symmetries are restored, occurs at very high Reynolds numbers, typically in the thousands, rendering challenging study of turbulence (both experimentally where flow systems of enormous size and/or high velocity are required and computationally where unconventionally high resolution in the numerical integration of the Navier-Stokes equation is needed). It is thus desirable that fluid turbulence can emerge in physical flows of relatively low Reynolds numbers.

The routes to turbulence in different flow systems can be different. For three-dimensional flows described by the Navier-Stokes equations, there are four main routes^5^,^6^: (a) quasi-periodicity (with two frequencies on two tori) and phase locking, (b) subharmonic (period doubling) bifurcations, (c) three frequencies (on two or three tori), and (d) intermittency. An example is that, in the Couette flow system with a rotating inner cylinder, fully developed turbulence can occur after a sequence of bifurcations^1^. In fact, the emergence of fully developed turbulence depends not only on the Reynolds number, but also on the particular characteristics of the underlying flow. For example, it was observed experimentally^7^ that the flow of a sufficiently elastic polymer solution in a small volume can become irregular even at low velocity. In combination with the high viscosity, the corresponding Reynolds number can be very low - on the order of unity. The irregular flow has all the key features of fully developed turbulence, i.e., it exhibits a broad range of spatial and temporal scales.

To understand and “control” turbulence^8^,^9^ is of great interest. Typically, the energy dissipation associated with a turbulent flow can be much larger than that with laminar flows. This can have economical impacts. For example, in oil pipelines the pressure required to pump a turbulent fluid is typically up to thirty times larger than that which would be necessary for laminar flows. To keep the flow laminar for as high values of the Reynolds number as possible, it is common to add polymers into the flow in oil pipelines. Similarly, one can consider ferrofluids in closed circular systems and use an applied magnetic field to keep the flow laminar. There were pioneering experimental^10^,^11^ and computational^11^,^12^ works on turbulence in ferrofluidic flows. Studying transition to turbulence in non-Newton fluid systems has thus been a topic of continuous interest.

In this paper, we investigate the Taylor-Couette flow^13^ in finite systems (e.g., aspect ratio Γ = 20) where a rotating *ferrofluid*^14^ is confined by axial end walls, i.e., non-rotating lids, in the presence of an external magnetic field. The classic Taylor-Couette flow of non-magnetic fluid has been a computational^15^,^16^ and experimental^17^,^18^,^19^ paradigm to investigate a variety of nonlinear and complex dynamical phenomena, including transition to turbulence at high Reynolds numbers^20^. The system we study consists of two independently rotating, concentric cylinders with viscous ferrofluid filled in between, which has embedded within itself artificially dispersed magnetized nanoparticles. In the absence of any external magnetic field, the magnetic moments of the nanoparticles are randomly oriented, leading to zero net magnetization for the entire fluid. In this case, the magnetized nanoparticles have little effect on the physical properties of the fluid such as density and viscosity. However, when a transverse magnetic field is applied, the physical properties of the fluid can be significantly modified^14^,^21^, leading to drastic changes in the underlying hydrodynamics. For example, for the system of rotating ferrofluid^14^, an external magnetic field can stabilize regular dynamical states^22^,^23^,^24^,^25^ and induce dramatic changes in the flow topology^22^,^23^,^26^. (In fact, the effects of magnetic field are particularly important for geophysical flows^27^,^28^,^29^,^30^). In general, the magnetic field can be used effectively as a control or bifurcation parameter of the system, whose change can lead to characteristically distinct types of hydrodynamical behaviors^22^,^23^,^24^,^25^. In this regard, transition to turbulence in magnetohydrodynamical (MHD) flows with current-driven instabilities of helical fields has been investigated^31^. There is also a large body of literature on MHD dynamics in Taylor-Couette flows^32^,^33^,^34^,^35^. Existing works on rotating ferrofluids^22^,^23^,^24^,^36^,^37^,^38^, however, are mostly concerned with steady *time-independent* flows. Time-dependent ferrofluid flows have been investigated only recently but in the non-turbulent regime^39^.

The interaction between ferrofluid and magnetic field leads to additional terms in the Navier-Stokes equation^22^,^25^,^37^. Our extensive computations reveal a sequence of transitions leading to time-dependent flow solutions such as standing waves with periodic or quasiperiodic oscillations, and turbulence. Surprisingly, we find that turbulence can occur for Reynolds numbers at least one order of magnitude smaller than those required for turbulence to arise in conventional fluids. The occurrence of turbulence is ascertained by carrying out a detailed investigation of the transitions between flow states and by examining the characteristics of the physical quantities such as the energy, the wave number, and the angular momentum. We also find that, for the Taylor-Couette ferrofluidic flow, the onset of turbulence can be determined accurately, in contrast to classical fluid turbulence where such a determination is typically qualitative and involves a high degree of uncertainty^2^. Our findings have the following implications:

- Revisiting and exploring the field of turbulence in ferrofluids can be rewarding, which has become feasible due to progress in computational fluid dynamics.
- In Taylor-Couette ferrofluidic flow the critical magnetic field strength for the onset of turbulence can be pinned down precisely, potentially leading to deeper insights into the physical and dynamical origins of the transition.
- Turbulence in Taylor-Couette ferrofluidic flows can be controlled via an external magnetic field.

In spite of the appealing features of ferrofluid flows, from the experimental point of view there are disadvantages to investigate turbulence in ferrofluids. For example, these fluids are opaque, rendering inapplicable standard methods of laser Doppler anemometry. It is necessary to employ ultrasonic Doppler velocimetry, but this requires rather bulky probes^40^. In addition, one needs tracer particles in the micrometer regime, which unavoidably create magnetic holes. The holes carry induced dipole moments that self-assemble to chains in direction of the applied magnetic field. The chains may dramatically change the properties of the flow. Such difficulties must be overcome in order to fully exploit ferrofluid for turbulence research.

## Results

### Ferrohydrodynamical equation of motion

Consider a Taylor-Couette system consisting of two concentric, independently rotating cylinders with an incompressible, isothermal, homogeneous, mono-dispersed ferrofluid of kinematic viscosity *ν* and density *ρ* within the annular gap. The inner and outer cylinders of radii *R*_1_ and *R*_2_ rotate at the angular speeds *ω*_1_ and *ω*_2_, respectively. The top and bottom end-walls^41^ are stationary and are at distance Γ(*R*_2_ − *R*_1_) apart, where Γ is the non-dimensionalized aspect ratio. The system can be described in the cylindrical coordinate system (*r*, *θ*, *z*) by the velocity field (*u*_*r*_, *u*_*θ*_, *u*_*z*_) and the corresponding vorticity **∇ ×*u* = (*ξ*, *η*, *ζ***). We set the radius ratio of the cylinders and the parameter Γ to typical values in experiments, e.g., *R*_1_/*R*_2_ = 0.5 and Γ = 20. A homogeneous magnetic field of strength *H*_*x*_ is applied in the transverse *x*-direction (*x* = *r* cos*θ*). The gap width *d* = *R*_2_ − *R*_1_ can be chosen as the length scale and the diffusion time *d*^2^/*ν* serves as the time scale. The pressure can be normalized by *ρν*^2^/*d*^2^, and the magnetic field **H** and the magnetization M by 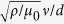 where *μ*_0_ is the magnetic permeability of free space. We then obtain the following non-dimensionalized equations governing the flow dynamics^25^,^42^:


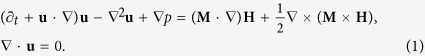


The boundary conditions on the cylinders are *u*(*r*_1_, *θ*, *z*) = (0, *Re*_1_, 0) and u(*r*_2_, *θ*, *z*) = (0, *Re*_2_, 0), where the inner and outer Reynolds numbers are *Re*_1_ = *ω*_1_*r*_1_*d*/*ν* and *Re*_2_ = *ω*_2_*r*_2_*d*/*ν*, respectively, and *r*_1_ = *R*_1_/(*R*_2_ − *R*_1_) and *r*_2_ = *R*_2_/(*R*_2_ − *R*_1_) are the non-dimensionalized inner and outer cylinder radii, respectively. To be concrete, we fix the Reynolds numbers at *Re*_1_ = 100 and *Re*_2_ = −150 so that the rotation ratio *β* = *Re*_2_/*Re*_1_ of the cylinders is −3/2.

We need to solve Eq. (1) together with an equation that describes the magnetization of the ferrofluid. Using the equilibrium magnetization of an unperturbed state where homogeneously magnetized ferrofluid is at rest and the mean magnetic moment is orientated in the direction of the magnetic field, we obtain M^eq^ = χH. The magnetic susceptibility χ of the ferrofluid can be approximated by using the Langevin’s formula^43^, where we set the initial value of χ to be 0.9 and use a linear magnetization law. The ferrofluid studied corresponds to APG933^44^. The near equilibrium approximations of Niklas^37^,^45^ are small ||M − M^eq^|| and small magnetic relaxation time *τ*: 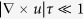 Using these approximations, one can obtain^25^ the following equation:


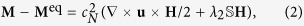


where


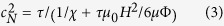


is the Niklas coefficient^37^, *μ* is the dynamic viscosity, *μ*_0_ is the vacuum permeability, Φ is the volume fraction of the magnetic material, 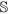 is the symmetric component of the velocity gradient tensor, and λ_2_ is the material-dependent transport coefficient^42^. We choose λ_2_ = 2, which corresponds to strong particle-particle interaction and chain formation of the ferrofluid^42^.

Using Eq. (2), we can eliminate the magnetization from Eq. (1) to obtain the following ferrohydrodynamical equation of motion^25^,^42^:


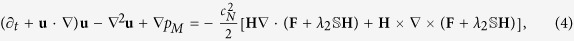


where **F** = (∇ × **u**/2) × **H** and *p*_*M*_ is the dynamic pressure incorporating all magnetic terms that can be expressed as gradients. To the leading order, the internal magnetic field in the ferrofluid can be approximated as being equal to the externally imposed field^24^, which is reasonable for obtaining dynamical solutions of the magnetically driven fluid motion. Equation (4) can then be simplified as


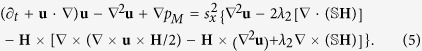


This way, the effect of the magnetic field and the magnetic properties of the ferrofluid on the velocity field can be characterized by a single parameter, the magnetic field or the Niklas parameter^37^,


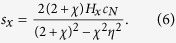


### Transition to turbulence in ferrofluids

**Figure 1:**
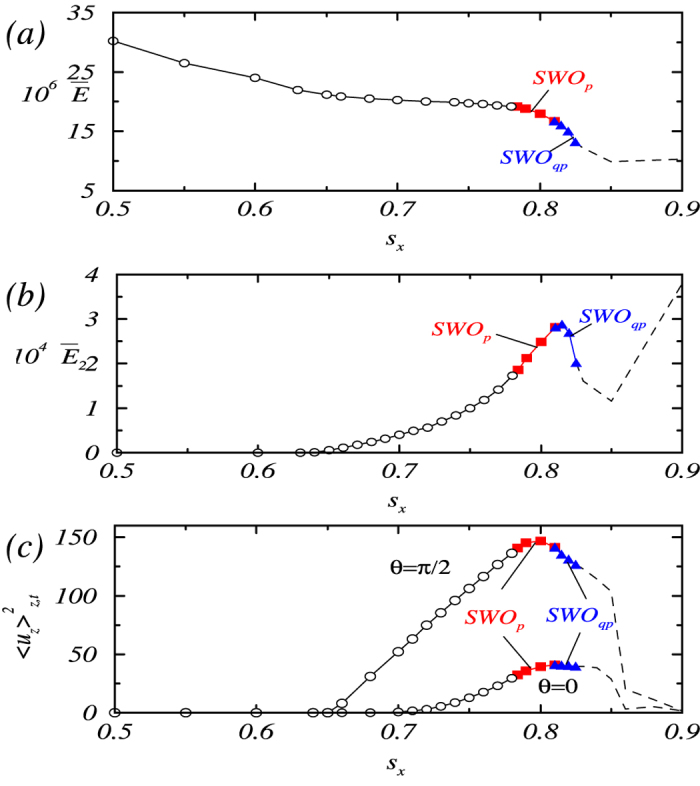
Distinct dynamical states for different values of the Niklas parameter *s*_*x*_: (a) time-averaged modal kinetic energy, (**b**) its *m* = 2 contribution, and (**c**) spatiotemporally averaged axial flow field at midgap for *θ* = 0 and *θ* = *π*/2. Open and filled symbols are for steady-state and time-dependent solutions, respectively. With increasing *s*_*x*_ the standing waves with axial oscillations appear first as periodic flow (SWO_*p*_) and later as quasiperiodic flow (SWO_*qp*_). For eye guidance, the dashed line sketches the variation of the corresponding quantities (time-averaged for fixed value of *s*_*x*_) with increasing Niklas parameter *s*_*x*_ in the turbulent regime.

**Figure 2:**
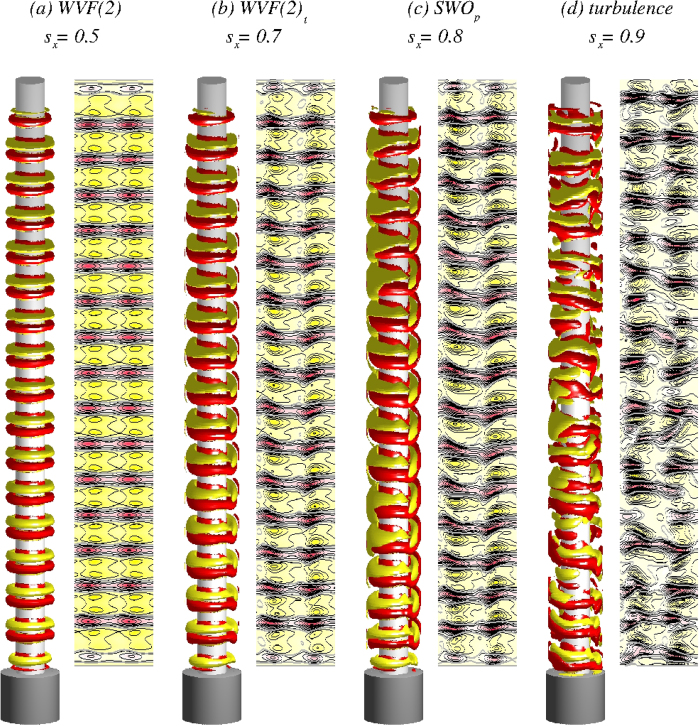
(a–d) For four values of *s*_*x*_ corresponding to wavy vortices with a two-fold symmetry with a “two-belly” structure (WVF(2))^22^, somewhat tilting pattern (WVF(2)_*t*_), time periodic standing waves with axial oscillations (SWO_*p*_), and turbulence regimes, respectively, isosurfaces of azimuthal vorticity *η* = *∂*_*z*_*u*_*r*_ − ∂_*r*_*u*_*z*_ and contours of the radial velocity u_r_(*θ,z*) on an unrolled cylindrical surface in the annulus at midgap. Red (dark gray) and yellow (light gray) colors denote *η* = ±100 for isosurfaces, and inflow and outflow for contour plots, respectively. While the patterns in (**a**,**b**) are stationary, the ones in (**c**,**d**) are snapshots due to time dependence of the corresponding flow.

We first present a sequence of transitions with the magnetic parameter *s*_*x*_, eventually leading to turbulence. For 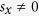, the rotating ferrofluid flow is intrinsically three-dimensional^22^,^23^,^24^,^25^ with increased complexity as *s*_*x*_ is increased from zero. Fig. 1(a–c) show, respectively, three key quantities versus *s*_*x*_: the time-averaged modal kinetic energy defined as


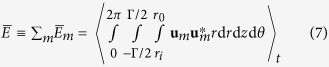


its *m* = 2 contribution 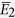, and the spatiotemporally averaged axial flow field 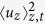 at midgap for *θ* = 0 (along the directions of the magnetic field) and *θ* = *π*/2 (perpendicular to the field). We see that, for *s*_*x*_ < 0.784, the flow is *time-independent*. In particular, for 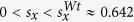, the flow patterns show wavy vortices with a two-fold symmetry^22^, denoted as WVF(2), which appears in Fig. 2(a) as a “two-belly” structure. As *s*_*x*_ is increa*s*ed through 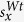, this symmetry is broken and a somewhat tilting pattern in the wavy-vortex structure emerges [denoted as WVF(2)_*t*_], as shown in Fig. 2(b). The intuitive reason is that the magnetic force is downward for fluid flow near the annulus and upward where the flow exits, resulting in a split in 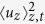 for *θ* = 0 and *π/*2. As *s*_*x*_ is increa*s*ed through the critical point 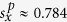, the flow becomes *time-dependent*. For 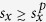, the flow is time periodic (limit cycle), corresponding to standing waves with axial oscillations of characteristic frequency *ω*_1_, which is denoted as SWO_*p*_. Near the onset of SWO_*p*_, the average kinetic energy values 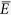 and 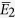 are unaffected, as can be seen from Fig. 1(a,b), respectively. For 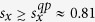, the periodic solution becomes unstable due to the emergence of a second incommensurate frequency *ω*_2_ (≈*ω*_1_/5) associated with defect propagation through the annulus in the axial direction, leading to transition to quasiperiodic flow (denoted as SWO_*qp*_). [See movie files movie1.avi, movie2.avi, movie3.avi and movie4.avi in Supplementary Materials (SMs), where the additional frequency can be identified visually in the defect starting from the top of the oscillating region, propagating downward toward the bottom, and vanishing there.] While 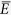 continues to decrease through this transition, both 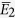 and 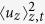 reach their respective maxima at the transition point. As *s*_*x*_ is increased further, the flow becomes more complex. Onset of turbulence occurs for 
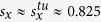
, where globally the flow starts to rotate in the azimuthal direction, as shown in Fig. 2(d) (see also movies movie5.avi, movie6.avi and movie7.avi in SMs). The observed route to turbulence coincides completely with that established previously^1^,^46^ for conventional fluid at high Reynolds numbers.

### Wave structure of the flows

**Figure 3:**
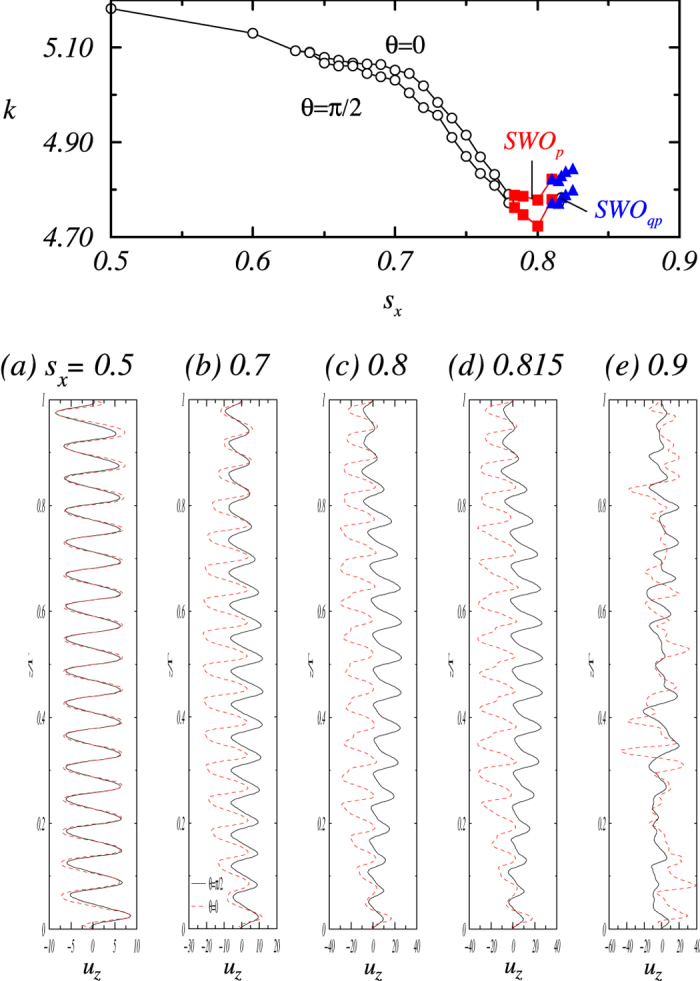
Variation with *s*_*x*_ of the axial wave number *k* in the directions along (*θ* = 0) and perpendicular to (*θ* = *π*/2) the magnetic field. (**a**–**e**) Snapshots of the axial velocity *u*_*z*_ for *θ* = 0 (dashed lines) and *θ* = *π*/2 (solid lines) in the annulus at the midgap location for five different values of *s*_*x*_. Panels (**a**) and (**b**) present the stationary states WVF(2) and WVF(2)_*t*_, respectively, while (**c**) and (**d**) correspond to periodic (SWO_*p*_) and quasiperiodic flow (SWO_*qp*_), and panel (**e**) illustrates a turbulent flow. The presented patterns are for *m* = 0 so that we can identify the largest contribution in the axial Fourier spectrum of (*u*_*z*_)_0_(*z*,*t*). In principle one can also identify *k* from the axial profiles in the figure that gives the axial wavelength λ and consequently the wavenumber *k* = 2*π*/λ. See also movie files movie8.avi, movie9.avi, movie2.avi, movie4.avi and movie7.avi in SMs.

We next analyze the wave structure of the flows to probe into the transition to turbulence in the low Reynolds-number regime. To get numerical solutions of the ferrohydrodynamical system, we solve the ferrohydrodynamical system by combining a finite-difference, time-explicit method of second order in (*r*, *z*) with Fourier spectral decomposition in *θ* (See Methods). The variables can be written as


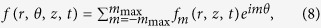


where *f* denotes any one of {*u*_*r*_, *u*_*θ*_, *u*_*z*_, *p*}. We then perform an axial Fourier analysis^22^ of the mode amplitudes of the radial velocity field (*u*_*r*_)_*m*_(*z*,*t*) at midgap to obtain the behavior of the wave number *k*, as shown in Fig. 3. Prior to the onset of turbulence, the number of vortices in the bulk fluid is invariant, irrespective of whether the flow is time-independent or time-dependent. However, due to the symmetry breaking induced by the magnetic field, the spatial structures of the flows can differ significantly. The top panel of Fig. 3 shows that the axial wave number *k* begins to split into two branches at 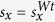, where *k* is enhanced and reduced as the field enters into and exits perpendicularly from the annulus, respectively, and there are dramatic changes in the flow profiles in which the dynamics tend to focus on the *θ* ≈ 0 region. As *s*_*x*_ is increased further, *k* in the bulk decreases with nearly constant difference between the cases of *θ* = 0 and *θ* = *π*/2. However, the kinetic energies and the axial wave numbers are nearly unaffected when the flow becomes time-dependent as *s*_*x*_ is increased through 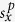. As the quasiperiodic regime is reached, the energies and the axial wave numbers begin to change where, as shown in Fig. 3, the wave numbers for *θ* = 0 and *θ* = *π*/2 are minimized at *s*_*x*_ about 0.8. For both the periodic and quasiperiodic regimes, there is little variation in *k* and only the vortex pair surrounding the oscillatory region expands and shrinks periodically. Especially, in the periodic regime the flow profile at *θ* = *π*/2 exhibits little dependence on *s*_*x*_ except for an increasing amplitude, but in the quasiperiodic regime the flow shows a strong dependence on *s*_*x*_, due to the emergence of the second frequency. As 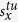 is passed, the flow starts to *rotate* and the split in the axial wave numbers in the directions parallel and perpendicular to the magnetic field vanishes. In fact, as turbulence sets in no distinct wave numbers can be identified, as shown in Figs. 2 and 3(e) (see also movie file movie7.avi in SMs).

### Behavior of the angular momentum

**Figure 4:**
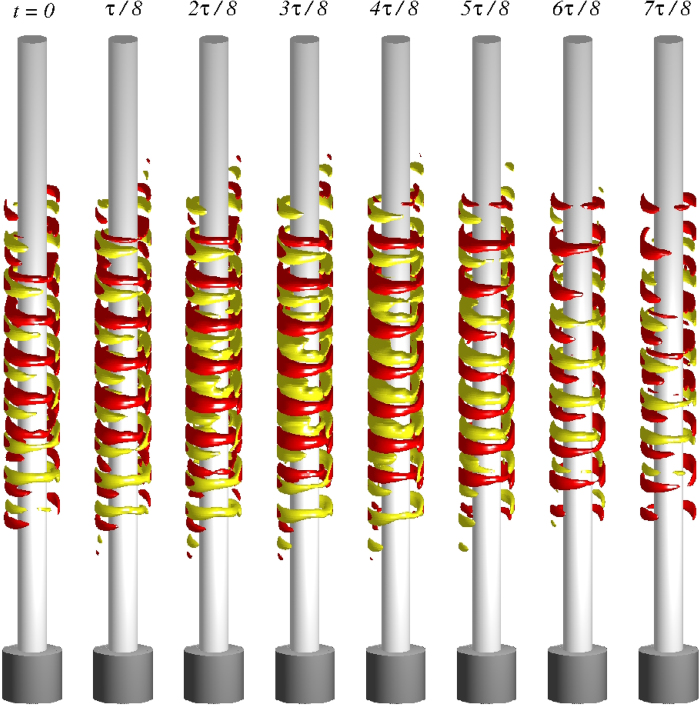
For *s*_*x*_ = 0.8 (periodic regime), isosurfaces of the relative angular momentum 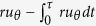 at eight time instants in one period of oscillation, where *τ* ≈ 0.058 and the isolevels are *ru*_*θ*_ = ±5 (see also movie files movie10.avi, movie11.avi, and movie12.avi in SMs).

We now examine the angular momentum in relation to transition to turbulence. Figure 4 shows, for flows in the periodic regime, the isosurfaces of differences in the angular momentum *ru*_*θ*_ between the full flow and its long-time averaged value, namely, . Onset of the periodic regime can be identified by the occurrence of periodic oscillations (up and down) of a single outward directed jet of angular momentum *ru*_*θ*_ (see movie files movie2.avi and movie1.avi in SMs). From Fig. 4, we see that, while the full flow pattern in the periodic regime exhibits little variation (see movie files movie10.avi and movie11.avi in SMs), there is relatively large variation in the behavior of the angular momentum (see movie12.avi in SMs). For example, there is a downward flow from *θ* = 0 and an upperward flow from *θ* = *π*, with opposite angular-momentum values. In the middle of the system where the effects of the Ekman boundary layers are minimal, the difference in the angular momentum reaches maximum. As the system enters into the quasiperiodic regime, the appearance of the incommensurate frequency *ω*_2_ signifies a kind of defects in the propagation pattern of the flow. As the flow begins to rotate in the azimuthal direction, turbulence sets in.

### Low Reynolds number turbulence

**Figure 5:**
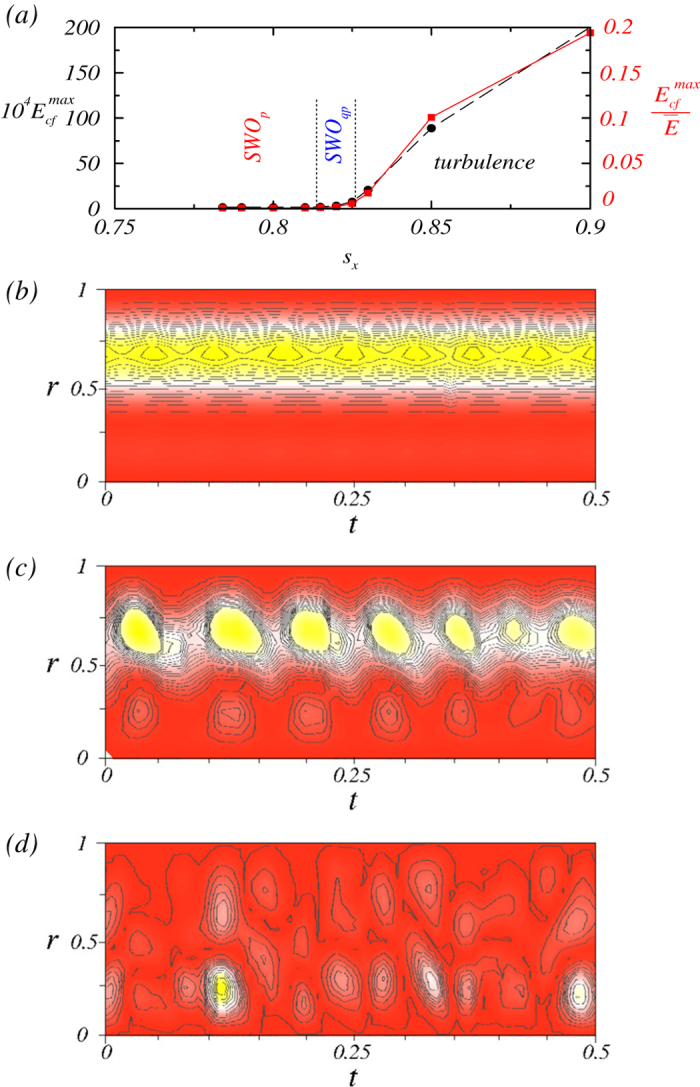
(a) Unnormalized and normalized (by the total kinetic energy) maximum cross-flow energy 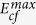 versus the Niklas parameter *s*_*x*_, where 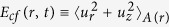 is averaged over the surface of the concentric cylinder. (**b**-**d**) Spatiotemporal evolutions of *E*_*cf*_(*r*,*t*) for *s*_*x*_ = 0.8, 0.82, and 0.9, corresponding to periodic, quasiperiodic, and turbulent regimes, respectively. Red (yellow) color indicates high (low) energy values.

A challenging issue in the study of turbulence is precise determination of its onset as a system parameter, e.g., the Reynolds number, is changed. For the type of low-Reynolds number turbulence in ferrofluid uncovered in this paper, this can actually be achieved. In particular, the transition to turbulence coincides with the onset of the azimuthal rotation from the quasiperiodic flow. To illustrate this, we consider the cross-flow energy *E*_*cf*_(*r*, *t*), a commonly used indicator for onset of turbulence in the Taylor-Couette system^47^, which is the instantaneous energy associated with the transverse velocity component at radial distance *r*. Small (large) values of *E*_*cf*_ indicate laminar (turbulent) flows. We find that, the maximum value of *E*_*cf*_(*r*, *t*), denoted by 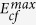, assumes near zero values for 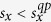 but it increases dramatically as *s*_*x*_ is increased through 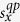, as shown in Fig. 5(a). In this figure we also plot the scaled maximum cross-flow energy value, 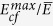, versus *s*_*x*_, and we observe that 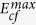 and 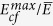 exhibit essentially the same behavior as the system passes through the onset of turbulence. Examples of the spatiotemporal evolution of *E*_*cf*_(*r*,*t*) are shown in Fig. 5(b–d) for periodic, quasiperiodic, and turbulent regimes, respectively. We observe regular patterns in the former two regimes, but no apparent patterns in the turbulent regime.

## Conclusions

**Figure 6:**
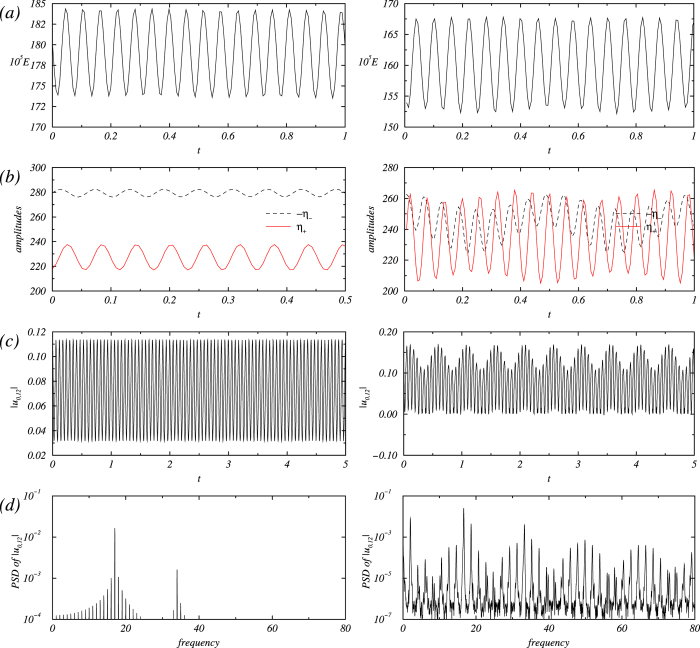
For two values of the magnetic parameters: *s*_*x*_ = 0.8 (SWO*_p_*, left column) and *s*_*x*_ = 0.815 (SWO*_qp_*, right column), time series of (a) modal kinetic energy E, (b) azimuthal vorticities *η*+ and − *η*− on the inner cylinder at two points symmetrically displaced about the midplane: *η*± = *η*(*r*_*i*_,0,±Γ/4,*t*)), (c) radial flow amplitudes |*u*_0,12_|, and (d) its corresponding power spectral density (PSD). The Reynolds numbers are *Re*_1_ = 100 and *Re*_2_ = −150.

**Figure 7:**
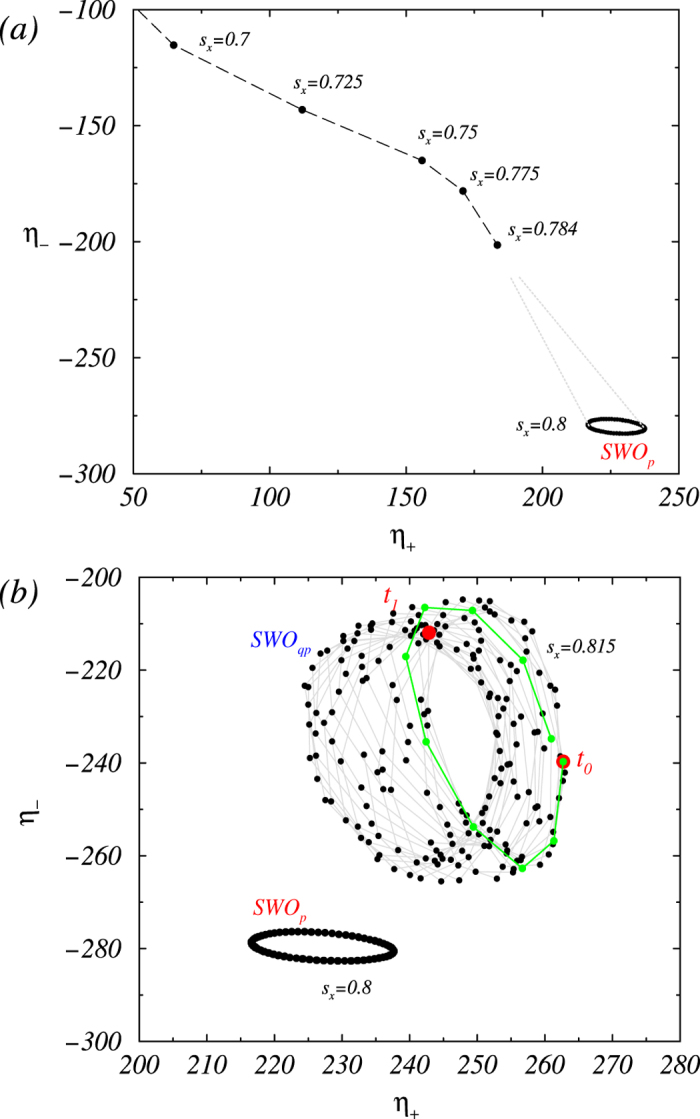
(a) Phase portraits of steady and time dependent limit-cycle solutions for different values of *s*_*x*_. The dotted bright gray lines are for eye guidance, which indicate how the limit-cycle (SWO_*p*_) evolve out of the fixed point solution. (**b**) Phase portraits of an extracted 2-torus associated with the quasiperiodic ate SWO_*qp*_. The two thick red points *t*_0_ and *t*_1_ are one diffusion time apart, during which the trajectory path (gray line with black points) circulates the 2-torus about 13 times. One of the nearly closed surrounding circles, which lasts about 0.077 diffusions time, is represented by the green colored part of the path starting at *t*_0_ and ending at *t*_1_.

We investigate the route to turbulence in the Taylor-Couette ferrofluid flow driven by a magnetic field transverse to the symmetric axis of the system. We find that the ferrofluidic flow can exhibit axial oscillations but not rotations in the azimuthal direction in the parameter regime where regular dynamics arise, and turbulence can occur generically for low values of Reynolds number. We elucidate the bifurcation sequence prior to onset of turbulence in detail. For this we considered either global and local quantities as exemplary presented in Figs. 6 and 7. For fixed (low) Reynolds number and small values of the magnetic parameter, the system exhibits a stationary fixed point. As the parameter is increased, the fixed point loses stability through a supercritical Hopf bifurcation, giving rise to a periodic limit cycle that becomes unstable through another Hopf bifurcation. At this bifurcation, a second incommensurate frequency emerges, leading to a quasiperiodic solution. As the magnetic parameter is increased further, there is a transition directly from quasiperiodic motion to turbulence. We find that turbulence can generically arise for low values of the Reynolds number. This is substantiated by extensive computations of the underlying ferrohydrodynamical equation through a detailed investigation of transitions in the flow structure by using a number of physical quantities. An implication lies in the perspective of controlled generation of turbulence through variations of the external magnetic field, making it possible to locate the onset of turbulence with high precision.

**Figure 8:**
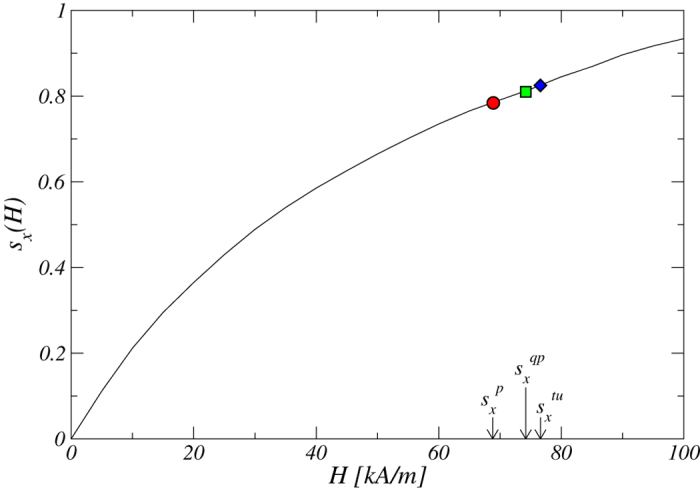
The Niklas parameter *s*_*x*_ versus the magnetic field strength H for ferrofluid APG933^44^. The symbols illustrate critical values for the onset of the limit cycle (periodic axial oscillation) for 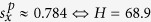, onset of 2-torus (quasiperiodic flow) for 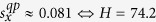, and finally onset of turbulence for 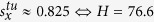.

We expect our findings to have values for experimental as well as theoretical investigation of turbulence. To substantiate this, we discuss the effect of different ferrofluid types and provide an estimate of the critical magnetic field strength required for onset of turbulence. The range of the magnetic parameter considered in this paper, i.e., 0 < *s*_*x*_ < 0.9, can in fact be realized in experimental studies of ferrofluids. Our computationally determined value of turbulence onset is 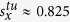, which corresponds to the magnetic field strength of about *H* = 76.6 [kA/m] [see Fig. 8 in Methods, *s*_*x*_(*H*) as function of the magnetic field *H*.] In our simulations we use the typical setting of counter-rotating cylinders with Reynolds number *Re*_1_ = 100 and *Re*_2_ = −150. The corresponding frequencies depend on the system setup and can be calculated from the given system parameters. In fact, the relation demonstrated in Fig. 8 depends on the ferrofluid type. In our simulations we use APG933, a type of magnetite based ferrofluid. For different ferrofluids, for example Cobalt based ferrofluids^23^, the effects due to magnetic fields can be “stronger,” meaning that similar dynamical behaviors can occur but for weaker magnetic fields. Indeed, for a Cobalt base ferrofluid, the corresponding curve (to that in Fig. 8) is much steeper. For ferrofluid APG933 studied in this paper, we expect onset of turbulence to occur in Taylor-Couette flow systems for critical magnetic strength less than about 80 kA/m, which can be realized in experiments.

## Methods

### Numerical scheme for ferrodynamical equation

System (4) can be solved^22^,^24^,^25^ by combining a second-order finite-difference scheme in (*r*,*z*) with Fourier spectral decomposition in *θ* and (explicit) time splitting. The variables can be written as


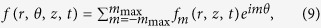


where *f* denotes one of {*u*_*r*_, *u*_*θ*_, *u*_*z*_, *p*}. For the parameter regimes considered in our preliminary study, the choice *m*_max_ = 10 provides adequate accuracy. We use uniform grids with spacing *δr* = *δz* = 0.05 and time-steps *δt* < 1/3800. For diagnostic purposes, we also evaluate the complex mode amplitudes *f*_*m*,*n*_(*r*, *t*) obtained from the Fourier decomposition in the axial direction 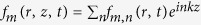, where *k* is the axial wavenumber.

### Detailed illustration of transitions prior to onset of turbulence

A method to ascertain the occurrence of turbulence is through examination of the sequence of transitions preceding the turbulence onset. The seminal work of Gollub and Swinney established that turbulence can arise directly from a quasiperiodic state^1^. Typical time series and power spectral densities from a detailed study of the type of flow states preceding turbulence onset are shown in Fig. 6, which is in complete agreement with the results in Ref. 1. The representative phase portraits for the periodic and quasiperiodic states are shown in Fig. 7.

### Relation between Niklas parameter *s*_*x*_(*H*) and the magnetic field strength *H*

Figure 8 shows, for ferrofluid APG933, the relation between *s*_*x*_(*H*) and *H*. From our numerically determined critical *s*_*x*_ value for onset of turbulence, we can determine the critical value of *H* using this relation.

## Additional Information

**How to cite this article**: Altmeyer, S. *et al*. Transition to turbulence in Taylor-Couette ferrofluidic flow. *Sci. Rep*. **5**, 10781; doi: 10.1038/srep10781 (2015).

## Supplementary Material

Supplementary Information

Supplementary Movie S1

Supplementary Movie S2

Supplementary Movie S3

Supplementary Movie S4

Supplementary Movie S5

Supplementary Movie S6

Supplementary Movie S7

Supplementary Movie S8

Supplementary Movie S9

Supplementary Movie S10

Supplementary Movie S11

Supplementary Movie S12
