# Supplementary material for: Transition to turbulence in Taylor-Couette ferrofluidic flow
Source: Sci Rep. 2015 Jun 12;5:10781. doi: 10.1038/srep10781 (PMC4464257; doi:10.1038/srep10781)
Supplement: Supplementary Information [file srep10781-s1.pdf]

# Supplementary Material to
# “Transition to turbulence in Taylor-Couette ferrofluidic flow”

Sebastian Altmeyer,[1,*] Younghae Do,[2,†] and Ying-Cheng Lai[3]

[1]*Institute of Science and Technology Austria (IST Austria), 3400 Klosterneuburg, Austria*
[2]*Department of Mathematics, KNU-Center for Nonlinear Dynamics, Kyungpook National University, Daegu, 702-701, South Korea*
[3]*School of Electrical, Computer and Energy Engineering, Arizona State University, Tempe, Arizona, 85287, USA*

(Dated: March 4, 2015)

**Legends for videos in SM**

- Movie 1:
  Movie 1 demonstrates SWO$_{qp}$ for $s_x = 0.82$ (quasiperiodic regime), isosurfaces of the azimuthal voticity $\eta = \partial_z u_r - \partial_r u_\theta = \pm 100$ (red: $\eta = 100$, yellow: $\eta = -100$).

- Movie 2:
  Movie 2 demonstrates SWO$_{qp}$ for $s_x = 0.82$ (quasiperiodic regime), axial velocity profiles $u_z$ for $\theta = 0$ (black) and $\theta = \pi/2$ (red) in the annulus at the midgap.

- Movie 3:
  Movie 3 demonstrates SWO$_{qp}$ for $s_x = 0.825$ (quasiperiodic regime), isosurfaces of the azimuthal voticity $\eta = \partial_z u_r - \partial_r u_\theta = \pm 100$ (red: $\eta = 100$, yellow: $\eta = -100$).

- Movie 4:
  Movie 4 demonstrates SWO$_{qp}$ for $s_x = 0.825$ (quasiperiodic regime), axial velocity profiles $u_z$ for $\theta = 0$ (black) and $\theta = \pi/2$ (red) in the annulus at the midgap.

- Movie 5:
  For $s_x = 0.9$ (turbulent regime), Movie 5 demonstrates isosurfaces of the angular momentum $ru_\theta = 80$.

- Movie 6:
  For $s_x = 0.9$ (turbulent regime), Movie 6 demonstrates isosurfaces of the azimuthal voticity $\eta = \partial_z u_r - \partial_r u_\theta = \pm 100$ (red: $\eta = 100$, yellow: $\eta = -100$).

- Movie 7:
  For $s_x = 0.9$ (turbulent regime), Movie 7 demonstrates axial velocity profiles $u_z$ for $\theta = 0$ (black) and $\theta = \pi/2$ (red) in the annulus at the midgap.

- Movie 8:
  Movie 8 demonstrates SWO$_p$ for $s_x = 0.8$ (periodic regime), axial velocity profiles $u_z$ for $\theta = 0$ (black) and $\theta = \pi/2$ (red) in the annulus at the midgap.

- Movie 9:
  Movie 9 demonstrates SWO$_{qp}$ for $s_x = 0.815$ (quasiperiodic regime), axial velocity profiles $u_z$ for $\theta = 0$ (black) and $\theta = \pi/2$ (red) in the annulus at the midgap.

- Movie 10:
  Movie 10 demonstrates SWO$_p$: for $s_x = 0.8$ (periodic regime), isosurfaces of the angular momentum $ru_\theta = 80$.

- Movie 11:
  Movie 11 demonstrates SWO$_p$ for $s_x = 0.8$ (periodic regime), isosurfaces of the azimuthal voticity $\eta = \partial_z u_r - \partial_r u_\theta = \pm 100$ (red: $\eta = 100$, yellow: $\eta = -100$).

- Movie 12:
  Movie 12 demonstrates SWO$_p$ for $s_x = 0.8$ (periodic regime), isosurfaces of the relative angular momentum $ru_\theta - \int_0^\tau ru_\theta dt = \pm 5$ (red: $\eta = 5$, yellow: $\eta = -5$).

* Electronic address: sebastian_altmeyer@t-online.de
† Electronic address: yhdo@knu.ac.kr
